# Validation of the *Edinburgh Postnatal Depression Scale* to assess maternal prenatal depression symptoms in Brazil: an item response theory analysis

**DOI:** 10.1590/0102-311XEN090525

**Published:** 2026-07-20

**Authors:** Márcia Leonardi Baldisserotto, Mariza Miranda Theme-Filha, Ana Claudia Santos Amaral, Maria Papaterra

**Affiliations:** 1 Instituto de Psicologia, Universidade Federal do Rio de Janeiro, Rio de Janeiro, Brasil.; 2 Escola Nacional de Saúde Pública Sergio Arouca, Fundação Oswaldo Cruz, Rio de Janeiro, Brasil.; 3 Instituto Nacional de Infectologia Evandro Chagas, Fundação Oswaldo Cruz, Rio de Janeiro, Brasil.

**Keywords:** Postnatal Depression, Maternal Health, Psychological Assessment, Depressão Pós-Natal, Saúde Materna, Avaliação Psicológica, Depresión Postnatal, Salud Materna, Evaluación Psicológica

## Abstract

The *Edinburgh Postnatal Depression Scale* (EPDS) is a widely used tool for assessing depressive symptoms in mothers during the perinatal period. Although it has been validated for postpartum depression screening in Brazil, no study has yet validated its use for prenatal depression screening. Therefore, this study aimed to validate the EPDS for assessing maternal prenatal depression symptoms in Brazil. The study tested the unidimensional structure of the EPDS using confirmatory factor analysis (CFA) and evaluated its reliability using composite reliability (CR). EPDS parameters were also analyzed using Samejima’s graded response model within an item response theory (IRT) framework. The results showed that the EPDS presented a unidimensional latent structure, with adequate construct validity (CFI = 0.974; TLI = 0.967; RMSEA = 0.072; 90%CI: 0.059-0.082) and reliability (CR = 0.91) for screening prenatal depression symptoms in Brazil. Additionally, the items showed satisfactory difficulty and discrimination parameters in IRT analyses. The total EPDS score provides more information than measurement error across a broad range of the latent trait (-2 to 4), indicating good accuracy and reliability. Overall, the EPDS seems to be an appropriate tool for screening maternal prenatal depression symptoms in Brazil.

## Resumo

A *Escala de Depressão Pós-natal de Edimburgo* (EPDS) é um instrumento amplamente utilizado para avaliar sintomas de depressão em mães durante o período perinatal. Embora seja validada para o rastreamento da depressão pós-parto no Brasil, não há estudo de validação para seu uso no rastreamento da depressão pré-natal. Portanto, este estudo teve como objetivo validar a EPDS para a avaliação de sintomas de depressão pré-natal no Brasil. O estudo testou a estrutura unidimensional da EPDS por meio da análise fatorial confirmatória (AFC) e avaliou sua confiabilidade utilizando a confiabilidade composta (CC). Os parâmetros da EPDS também foram analisados empregando o modelo de resposta graduada de Samejima junto à teoria da resposta ao item (TRI). Os resultados mostraram que a EPDS apresentou estrutura unidimensional com valores adequados de validade de construto (CFI = 0,974; TLI = 0,967; RMSEA = 0,072; IC90%: 0,059-0,082) e de confiabilidade (CC = 0,91) para o rastreamento de sintomas de depressão pré-natal no Brasil. Além disso, os itens do instrumento apresentaram bons parâmetros de dificuldade e discriminação nas análises de TRI. O escore total da EPDS fornece mais informação do que erro de medida em um amplo espectro do traço latente (-2 a 4), indicando boa precisão e confiabilidade. A EPDS demonstra ser uma ferramenta adequada para o rastreamento de sintomas de depressão pré-natal materna no Brasil.

## Resumen

La *Escala de Depresión Postnatal de Edimburgo* (EPDS) es un instrumento ampliamente utilizado para evaluar los síntomas depresivos en las madres durante el período perinatal. Aunque validada para el cribado de la depresión posparto en Brasil, no existe ningún estudio de validación para su uso en el cribado de la depresión prenatal. Por lo tanto, este estudio tuvo como objetivo validar la EPDS para la evaluación de los síntomas depresivos prenatales en Brasil. El estudio probó la estructura unidimensional de la EPDS mediante el análisis factorial confirmatorio (AFC) y evaluó su fiabilidad mediante la fiabilidad compuesta (FC). Los parámetros de la EPDS también se analizaron utilizando el Modelo de Respuesta Graduada de Samejima dentro del marco de la teoría de respuesta al ítem (TRI). Los resultados mostraron que la EPDS presentó una estructura unidimensional con valores adecuados de validez de constructo (CFI = 0,974; TLI = 0,967; RMSEA = 0,072; IC90%: 0,059-0,082) y fiabilidad (CC = 0,91) para el cribado de los síntomas de depresión prenatal en Brasil. Además, los ítems del instrumento mostraron buenos parámetros de dificultad y discriminación en los análisis de IRT. La puntuación total de la EPDS proporciona más información que el error de medición en un amplio espectro de rasgos latentes (de -2 a 4), lo que indica buena precisión y fiabilidad. La EPDS demuestra ser una herramienta adecuada para el cribado de síntomas de depresión prenatal materna en Brasil.

## Introduction

Maternal perinatal depression is a common mental health condition that occurs during both prenatal and postnatal periods ^1^. It can negatively affect the physical and mental health of both the mother and infant, and may also impact child development ^2^ ^,^ ^3^. Notably, prenatal depression is often associated with postnatal depression, as many women who experience postnatal depression begin to show symptoms during pregnancy. Therefore, early detection during pregnancy is critical ^4^. This requires reliable measuring instruments, such as self-report scales, that can identify women showing symptoms of this disorder early in pregnancy. Self-report scales are affordable and do not require extensive training, making them effective tools for screening maternal depression in early pregnancy.

Currently, the *Edinburgh Postnatal Depression Scale* (EPDS) is one of the most used tools for measuring maternal prenatal and postpartum depressive symptoms ^5^ ^,^ ^6^. The instrument was developed to assess symptoms of postnatal depression and was created by Cox et al. ^7^ in 1987. The scale consists of 10 self-report items, each rated on a scale from 0 to 3, indicating the frequency with which the woman experienced each symptom over the past seven days. The total score ranges from 0 to 30, with higher scores indicating a greater likelihood of depression symptoms ^7^. The EPDS has demonstrated good psychometric validity and reliability ^8^ ^,^ ^9^. Although there is evidence supporting a multidimensional factor model ^10^ ^,^ ^11^, the original model is unidimensional ^7^.

In 1990, Murray & Cox ^12^ examined the validity of the EPDS as a screening tool for depressive symptoms during pregnancy. The authors argued that the EPDS items are not specific to postnatal depression but can also capture depressive symptoms during pregnancy. Therefore, they concluded that no modifications to the original EPDS items are necessary for use in pregnant women. Murray & Cox found promising sensitivity and specificity for EPDS in pregnant women and subsequently recommended its use ^12^.

Studies on the psychometric properties of the EPDS for prenatal depression screening indicate good validity and reliability across countries ^6^ ^,^ ^13^ ^,^ ^14^ ^,^ ^15^. According to the available literature, both one-dimensional ^15^ and two-dimensional factorial models ^16^ have been proposed to describe the factor structure of the EPDS. However, no studies have examined the psychometric properties of the EPDS in pregnant women using item response theory (IRT).

In Brazil, the EPDS is commonly used to assess symptoms of maternal prenatal and postnatal depression ^17^ ^,^ ^18^. However, while its validity has been established for screening postnatal depression ^19^ ^,^ ^20^, no study has validated the EPDS for screening prenatal depression symptoms. It remains unclear whether this instrument is reliable and valid for use in pregnant women. Hence, future research is required to investigate the psychometric properties of the EPDS in a sample of pregnant women and to verify its validity and reliability in the Brazilian context.

This study aimed to evaluate the construct validity and reliability of the EPDS for screening maternal prenatal depression symptoms in Brazil using classical test theory (CTT) and to analyze item discrimination (a) and difficulty (b) parameters using the IRT approach. The objective is to offer a reliable and valid tool for scientific research and clinical practice in Brazil, supporting healthcare professionals in identifying maternal prenatal depression symptoms in primary care settings.

## Method

### Study design, participants, and procedures

This research is part of a cohort conducted from February 2016 to November 2019 in two primary healthcare units in Rio de Janeiro, Brazil. A total of 513 pregnant women with low obstetric risk, less than 20 weeks of gestation, and aged 18 years or older were included in the sample. The EPDS was administered in-person by a trained interviewer using a structured questionnaire. The tool was applied at the healthcare units where the participants attended their prenatal appointments.

### Instruments

The EPDS was used to assess maternal perinatal depression symptoms during pregnancy. This instrument was developed by Cox et al. ^7^. This Brazilian Portuguese version of the tool, validated for the Brazilian population by Santos et al. ^19^, was employed. It consists of 10 items, with total scores ranging from 0 to 30; higher scores indicate a greater presence of depression symptoms ^7^.

Sociodemographic and obstetric data (age, marital status, educational attainment, employment status, and satisfaction with pregnancy) were obtained using a structured questionnaire administered during the first interview of the cohort study.

### Data analysis

Initial analyses were conducted to describe sociodemographic characteristics of the sample. Quantitative variables were presented as means and standard deviations, while categorical variables were expressed as percentages. The unidimensional latent trait model, originally proposed by Cox et al. ^7^, was tested using confirmatory factor analysis (CFA) with a robust weighted least squares estimator (weighted least squares mean and variance - WLSMV) for parameter estimation. Model fit assessed using the comparative fit index (CFI), Tucker-Lewis index (TLI), root mean square error of approximation (RMSEA), and standardized root mean square residual (SRMR). Good model fit was indicated by CFI and TLI values greater than 0.95, RMSEA values below 0.06, and SRMR values below 0.05. Additionally, a 90% confidence interval (90%CI) for RMSEA was calculated and considered adequate when the lower limit was close to 0 and the upper limit was below 0.08. Items with standardized factor loadings of at least 0.50 were considered acceptable for inclusion in the factorial model ^21^. The reliability of the EPDS was assessed using composite reliability (CR), with values greater than 0.7 considered adequate ^21^ ^,^ ^22^.

Subsequently, the EPDS items were analyzed using IRT. For this purpose, Samejima’s two-parameter graded response model (GRM) was applied ^23^ ^,^ ^24^. This model was used to estimate the difficulty (b) and discrimination (a) parameters for each item. In the GRM, the difficulty parameter (b), also referred to as the threshold or location parameter, represents the point along the latent trait (θ) - maternal prenatal depression symptoms continuum - at which a respondent has a 50% probability of endorsing a given response category or a higher one. Higher b values indicate more “difficult” categories, as they require higher levels of the underlying trait for endorsement ^23^ ^,^ ^24^. The discrimination parameter (a) was interpreted using the following classification: 0 = no discrimination; 0.01-0.34 = very low; 0.35-0.64 = low; 0.65-1.34 = moderate; 1.35-1.69 = high; and > 1.7 = very high ^25^.

The precision of the EPDS total score was also evaluated with a graphical analysis of the test information function (TIF) and its corresponding standard error (SE), estimated using the IRT model. The TIF quantifies the amount of information the instrument provides across different levels of the latent trait (θ), with higher values indicating greater measurement precision. The SE is inversely related to the TIF; thus, higher information corresponds to lower measurement error. The assessment of the TIF curve focused on three aspects: the location of the peak, indicating the latent trait level at which the instrument achieves maximum precision; the height of the curve, reflecting the magnitude of information and overall precision; and the width of the curve, representing the range of θ values over which the instrument yields reliable measurement. A high and broad TIF curve indicates strong precision across a wide range of the latent trait, while a narrow curve suggests precision limited to a specific region. The SE curve was analyzed alongside the TIF to identify regions of higher and lower measurement accuracy. SE values below 0.30 were interpreted as indicating excellent precision, whereas values above 0.50 indicated reduced reliability across that range of θ. The interpretation of both curves was used to determine whether the instrument was most informative at low, medium, or high levels of the latent construct ^23^ ^,^ ^24^ ^,^ ^25^ ^,^ ^26^.

Statistical analyses were performed using R, version 3.5.1 (http://www.r-project.org), and Mplus, version 8 (https://www.statmodel.com/).

### Ethical issues

This study was conducted in accordance with *Resolution n. 196/1996* of the Brazilian National Health Council, which establishes guidelines for research involving human beings. Ethical approval was granted by the Research Ethics Committee of the Sergio Arouca National School of Public Health, Oswaldo Cruz Foundation (CAAE 21982613.6.0000.5240). All participants received and signed an informed consent form.

## Results

Most women included in the study had 10-13 years of schooling (50.7%), lived with a partner (77.5%), self-identified their ethnicity as Mixed-race (49.3%), and had no paid employment (53.4%). The mean age was 26.2 years (standard deviation - SD = 5.86), and the mean EPDS score was 6.6 (SD = 2.23).

The unidimensional latent trait model of the EPDS showed good values for CFI (0.974), TLI (0.967), and SRMR (0.041), and regular value for RMSEA (0.072, 90%CI: 0.059-0.082). Factor loadings ranged from 0.575 (EPDS6) to 0.865 (EPDS9). The EPDS demonstrated adequate reliability, with a CR of 0.91. These results indicate the robustness of the unidimensional factor model (Table 1).

**Table 1:** Confirmatory factor analysis (CFA) and reliability of the *Edinburgh Postnatal Depression Scale* (EPDS): standardized factor loadings, measurement errors, and model fit estimates.

Item	CFA	
	λi	δi
EPDS1 - I have been able to laugh and see the funny side of things	0.724	0.524
EPDS2 - I have looked forward with enjoyment to things	0.689	0.475
EPDS3 - I have blamed myself unnecessarily when things went wrong	0.651	0.424
EPDS4 - I have been anxious or worried for no good reason	0.602	0.362
EPDS5 - I have felt scared or panicky for no very good reason	0.684	0.468
EPDS6 - Things have been getting on top of me	0.575	0.331
EPDS7 - I have been so unhappy that I have had difficulty sleeping	0.790	0.624
EPDS8 - I have felt sad or miserable	0.860	0.740
EPDS9 - I have been so unhappy that I have been crying	0.865	0.748
EPDS10 - The thought of harming myself has occurred to me	0.770	0.593
CR	0.91	
CFI/TLI	0.974/0.967	
RMSEA (90%CI)	0.072 (0.059-0.082)	
SRMR	0.041	

90%CI: 90% confidence intervals; δ: measurement error; λ: factor loading; CFI: comparative fit index; CR: composite reliability; RMSEA: root mean square error of approximation; SRMR: standardized root mean square residual; TLI: Tucker-Lewis index.

The items covered a wide range of the underlying trait, with threshold values (b) ranging from -0.458 (b1 for item EPDS3) to 2.782 (b3 for item EPDS10). Item EPDS3 demonstrated the lowest endorsement difficulty (b3 = 1.264), whereas item EPDS10 showed the highest (b3 = 2.782) (Table 2).

**Table 2:** Item difficulty (b) and discrimination (a) parameters of the *Edinburgh Postnatal Depression Scale* (EPDS) estimated using Samejima’s graded response model (GRM) - item response theory (IRT).

Item	a	b1	b2	b3
EPDS1	1.846	0.506	1.841	2.510
EPDS2	1.676	0.095	1.443	2.314
EPDS3	1.477	-0.458	-0.049	1.264
EPDS4	1.209	-0.421	0.538	1.986
EPDS5	1.563	0.756	1.149	2.198
EPDS6	1.221	-0.364	0.470	1.949
EPDS7	2.433	0.938	1.179	1.765
EPDS8	3.134	0.319	0.905	1.305
EPDS9	3.261	0.433	1.374	1.738
EPDS10	2.444	1.368	1.739	2.782

The results indicate that the EPDS items exhibited varying levels of discrimination. Two items showed moderate discrimination (EPDS4 = 1.209; EPDS6 = 1.221), three showed high discrimination (EPDS2 = 1.676; EPDS3 = 1.477; EPDS5 = 1.563), and five showed very high discrimination (EPDS1 = 1.846; EPDS7 = 2.43; EPDS8 = 3.134; EPDS9 = 3.261; EPDS10 = 2.444). Overall, discrimination parameters ranged from moderate (EPDS4 = 1.209) to very high (EPDS9 = 3.261) (Table 2).

**Figure 1:**
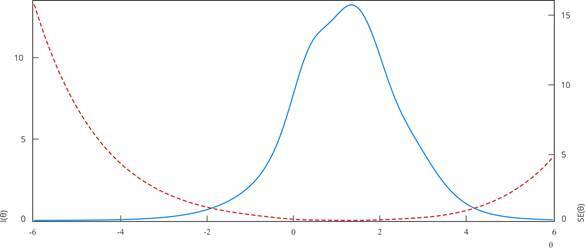
Test information function and standard error curves for the *Edinburgh Postnatal Depression Scale* (EPDS).

Figure 1 presents the TIF and the corresponding SE for the EPDS total score. As shown, the EPDS provides adequate measurement precision, with lower SE values across the latent continuum of maternal prenatal depressive symptoms, particularly at θ levels ranging from 2 to 4. Within the range 0-4, SE values < 0.30 indicate very good measurement precision. Additionally, the peak of the curve is located at θ > 1, suggesting that the EPDS is more sensitive to higher levels of prenatal depression symptoms.

I(θ): latent trait information function; SE(θ): standard error of the latent trait function; θ: latent trait (maternal prenatal depression symptoms).

## Discussion

The EPDS presented a unidimensional latent trait model to screen maternal prenatal depression symptoms in Brazil. Moreover, the items of the instrument showed satisfactory difficulty and discrimination parameters in the IRT analyses. The total EPDS score provided adequate measurement precision, with low measurement error across a broad range of the latent trait (-2 to 4), indicating good accuracy and reliability. Therefore, the results suggest that the EPDS is an adequate tool for screening maternal prenatal depression symptoms in Brazil.

Results demonstrating adequate psychometric properties of validity and reliability of the EPDS in pregnant women, assessed using CFA and CR, were consistent with the literature ^6^ ^,^ ^13^ ^,^ ^14^ ^,^ ^15^. Furthermore, the findings support the unidimensional factor model originally proposed by Cox et al. ^7^. However, some results could not be directly compared with the existing literature due to the lack of studies applying IRT to analyze EPDS items for screening maternal prenatal depression symptoms.

Regarding the discrimination parameter (a) of the EPDS items, most items showed high to very high discrimination. High discrimination indicates that the EPDS items can effectively differentiate between individuals with low and high levels of prenatal depression symptoms, which is essential for a mental health screening tool. As previously noted, this pattern of high item discrimination is important for the EDPS, as it is designed to assess symptoms of mental health disorder ^7^.

The EPDS items also demonstrated good variability in difficulty (b) levels, enabling the instrument to detect various levels of prenatal depression symptoms among pregnant women. Therefore, the set of items covers a broad spectrum of the underlying latent trait. The item with the highest endorsement difficulty was EPDS10 (b3 = 2.782): “The thought of harming myself has occurred to me”. This finding is consistent with the literature, as this item is associated with more severe perinatal depression symptoms ^7^ ^,^ ^12^.

In the IRT analysis of the total score, based on the TIF, the EPDS items covered a broad range of the latent trait of maternal prenatal depression symptoms. The instrument demonstrated adequate measurement precision, with lower measurement error across θ levels ranging from -2 to 4. Within the interval from 0 to 4, the EPDS showed very good accuracy, as indicated by the SE values. These findings suggest that the EPDS can accurately identify women with prenatal depression symptoms within this range of the latent trait. Additionally, the peak of the TIF curve at θ > 0 indicates greater measurement precision among individuals with higher levels of antenatal depressive symptoms ^23^ ^,^ ^24^ ^,^ ^25^. Consequently, the instrument appears more sensitive in detecting and differentiating individuals with moderate to severe symptoms, while showing lower precision for those with minimal or no symptoms. This pattern is adequate for screening instruments such as the EPDS, which are designed to identify individuals at risk for, or experiencing, clinically relevant antenatal depressive symptoms ^7^.

Despite making an important contribution to the analysis of the applicability of the EPDS for screening prenatal depression among pregnant women in Brazil, this study presents some limitations that may have influenced the results, especially in the CFA and CR analyses. The sample consisted of pregnant women from a low-income population with limited socioeconomic variability, which may have influenced validity and reliability findings. Conversely, this limitation is less likely to have influenced the IRT analyses, as these are less dependent on sample characteristics ^25^ ^,^ ^26^, representing a strength of this study.

Further studies evaluating the psychometric properties of the EPDS to assess maternal prenatal depression symptoms in Brazil are necessary. Future research should examine additional aspects of validity, including content and criterion validity, as well as assess reliability using alternative approaches, such as test-retest methods. Additionally, it is recommended to investigate the instrument in more diverse populations beyond those included in this study.

## Conclusion

This study found that the EPDS may be a reliable and valid tool for measuring maternal prenatal depression symptoms in Brazil. Therefore, it can be used in scientific research to advance knowledge in this area. Additionally, healthcare providers may use it in prenatal care to screen mothers who may be experiencing prenatal depression symptoms.

## Data Availability

The research data are available upon request to the corresponding author.
